# Characterizing ICU-like profiles of very old patients hospitalized in medical intermediate care units in France: A clustering analysis of a nationwide population-based study

**DOI:** 10.1016/j.aicoj.2026.100031

**Published:** 2026-01-30

**Authors:** Arthur Kassa-Sombo, Adrien Migeon, Lucile Godillon, Emeline Laurent, Leslie Grammatico-Guillon, Antoine Guillon

**Affiliations:** a Research Center for Respiratory Diseases (CEPR), INSERM U1100, University of Tours, Tours, France; b Department of Geriatrics, Tours University Hospital, Tours, France; c Epidemiology UnitEpiDcliC, Clinical Data Center, Department of Public Health, Tours University Hospital, France; d Research Unit EA1075 (Education, Ethics and Health), University of Tours, Tours, France; e Research Unit MAVIVH, INSERM U1259, Medical School, University of Tours, Tours, France; f Intensive Care Unit, Tours University Hospital, University of Tours, Tours, France

**Keywords:** Very old patient, Intermediate care unit

## Abstract

**Purpose:**

The population aging has a growing impact on intensive care. The admission policies for critically ill very old patients in intermediate care units (IMCUs) are unclear. This study aimed to identify clusters of very old patients hospitalized in IMCUs in France, and to compare “ICU-like” profiles among these patients with matched very old patients admitted to ICU patients, in order to better understand the factors influencing admission to an IMCU rather than an ICU.

**Methods:**

We conducted a nationwide study and examined all hospitalisations in IMCUs in France over a two-year period. We studied the utilization of medical IMCU for all critically ill patients ≥80 y.o and reported patient characteristics, including the Charlson comorbidity index and Hospital Frailty Risk Score. Latent class analysis identified distinct IMCU phenotypes. Clusters with high-use organ support (≥5%) and high mortality (≥40%) were considered “ICU-like.” Logistic regression compared “ICU-like” IMCU patients with propensity-matched ICU patients to identify factors associated with IMCU admission.

**Results:**

Among the 202,976 very old individuals hospitalized in IMCU, seven phenotypes were identified. The "ICU-like" population accounted for 11.6% of the cohort (*n* = 23,508). After propensity score matching, the multivariate logistic regression identified age as the strongest determinant to IMCU admission, with very old patients being significantly more likely to be admitted to the IMCU over the ICU. Comorbidity status was also an independent predictor for admission to IMCU. Frailty status showed only a marginal effect, and no association was detectable for sex.

**Conclusion:**

A substantial number of very old patients were admitted to IMCU despite meeting ICU-level criteria. Clearer admission criteria and objectives for IMCU care in older critically ill patients are urgently needed.

## Introduction

The population aging has a growing impact on intensive care. As a result, the proportion of very old patients in intensive care units (ICUs) has doubled over the past decade [1,2]. ICUs deliver the most advanced level of medical care within a hospital environment, particularly in terms of personnel and infrastructural assets. Intermediate care units (IMCUs) offer a level of care that bridges the gap between the high intensity of the ICU and the standard hospital wards [3,4]. Indeed, IMCUs are intended to treat patients whose physiological parameter instability requires equipment-based monitoring but only low-level organ support [5,6]. With the growing global challenge posed by the care of very old critically ill patients, their admission to IMCU increases. We previously demonstrated that nearly one-third of patients hospitalized in IMCU in France are aged 80 years and older [7]. However, the admission policies for critically ill very old patients in IMCU are unclear. An unknown proportion of critically ill very old patients are admitted to IMCU because ICU admission was denied. This ambiguity in admission policies raises critical questions about the appropriateness of care for very old patients [[8], [9], [10]]. The substantial heterogeneity previously described among very old patients hospitalized in the IMCU makes it difficult to reliably estimate the proportion of patients admitted with palliative support or following a decision to withdraw life-sustaining treatment [11].

We hypothesized that combinations of acute and geriatric characteristics, together with therapeutic interventions, define distinct phenotypes in the IMCU. We also anticipated that some patient clusters would exhibit frequent use of high-level organ support and elevated mortality, forming an ‘ICU-like’ subgroup within IMCU. This study aimed to identify clusters of very old patients hospitalized in IMCUs in France, and to compare ICU-like profiles among these patients with matched very old patients admitted to ICU patients, in order to better understand the factors influencing admission to an IMCU rather than an ICU.

## Methods

### Selection of participants

A retrospective cohort was built using data from the French Hospital Discharge Database (HDD), as previously described [7]. Briefly, we included all patients aged ≥80 years from the adult population hospitalized in France, using 80 years as the threshold for defining very old patients. Surgical critical care units, burn units, and organ transplant cases were excluded. Patients admitted to both the ICU and IMCU during the same hospital stay were classified in the ICU group; ICU admission was prioritized for classification purposes. Patients were included on a 2-year period from 2017 to 2018. To better understand the characteristics of included patients, we examined their comorbidities and frailty starting from 2014.

### Selection of indicator variable

For each hospital stay, we extracted data on age, sex, comorbidities, length of stay, and in-hospital death. The Charlson Comorbidity Index (CCI) was calculated from the international classification of diseases, 10th version (ICD-10) codes [12], and frailty risk was assessed using the validated Hospital Frailty Risk Score (HFRS) for older adults [13,14]. Admission reasons were based on the primary ICD-10 diagnosis in the IMCU or ICU and classified by organ system. Admission reasons were based on the primary ICD-10 diagnosis for the hospital stay in the IMCU or ICU and subsequently classified by organ system as neurological dysfunction, cardiac dysfunction/circulatory failure, respiratory organ dysfunction or other diagnoses (Supplementary Table S1). High-level organ supports, including inotropic or vasopressor support and invasive or non-invasive mechanical ventilation, were identified using French common procedure terminology codes (Supplementary Table S2). The Simplified Acute Physiology Score II (SAPS II) was obtained from hospital records in the French HDD [15]. We also collected data on total and unit-specific length of stay (IMCU or ICU), vital status at discharge and at one year.

### Latent Class Analysis (LCA)

LCA is a statistical procedure used to identify subgroups within populations that share certain characteristics (i.e. cluster) [16,17]. LCA assumes the existence of unobserved (latent) classes that explain patterns in observed responses, with responses being independent of each other once class membership is accounted for. The method estimates two key probabilities: the likelihood of an individual belonging to a specific latent class, and the probability of each class exhibiting certain response patterns. These estimates are then used to define and describe the unique dimensions and profiles of each cluster. In LCA, variables are exclusively categorical. Input information for the LCA were: age, comorbidities (assessed by Charlson comorbidity index), type of organ dysfunction, SAPSII, organ support, length of stay, death (see Supplemental Table S3 for more details). In this study, models were initiated with multiple sets of random starting values. The optimal number of classes was determined by comparing fit indices Bayesian Information Criterion (BIC), adjusted Bayesian Information Criterion (aBIC), Akaike Information Criterion (AIC), Lo-Mendell-Rubin ad hoc likelihood ratio test (LMRa), and entropy, as well as by considering the interpretability and parsimony of the solutions. The AIC, BIC and aBIC served as criteria for class number selection with lower value indicating a better trade-off between fit and complexity of model. Entropy measures the clarity of classification, and the separation of classes ranged from 0 to 1. An entropy of ≥0.80 indicates a well-defined classification with clear separation between classes. The LMRa test was used to assess whether a model with k classes provided a significantly better fit than a model with k-1 classes. LCA was performed using poLCA in R Version 4.3.2. The poLCAParallel, a reimplementation of poLCA, was used to speed up the running.

### Multiple Correspondence Analysis (MCA)

MCA is a method for exploring relationships between categorical variables by reducing data to a smaller number of dimensions. The variance in the data is summarized and explained by the principal axes, which capture the main sources of variability among the categorical variables. A graphical projection of the optimal number of clusters determined by the LCA was performed using MCA to display the distribution of individuals and illustrate the heterogeneity between clusters. Additionally, a spider chart was used to display the distribution of the variables included in the LCA within each cluster.

### Definition of “ICU-like” clusters

We hypothesized that one or more clusters in the IMCU would be characterized by frequent use of high-level organ support and elevated mortality. In the ICU, approximately 40% of patients received specific organ support and nearly 40% died during their stay [7]. For the IMCU, we defined a lower cut-off value for frequent use of high-level organ support lower than those observed in the ICU, assuming that treatment restrictions are more common in the IMCU. We arbitrarily considered the use of high-level organ support within a latent class to be significant when ≥ 5% of patients received it. For mortality, we considered it important in the IMCU when it was equal to or greater than that observed in the ICU (≥ 40%). In summary, latent classes with significant use of high-level organ support (≥5%) and high mortality (≥40%) were defined as “ICU-like” clusters.

### Propensity Score Matching (PSM)

In this study, a propensity score was estimated using a logistic regression model that included Simplified Acute Physiology Score II (SAPS II), markers of intensive care interventions and the type of organ dysfunction as predictors of an ICU profile. Individuals from different groups were then matched based on similar propensity scores using the nearest-neighbor algorithm with a caliper width of 0.005 on the logit of propensity score, without replacement. The quality of the propensity score was visually assessed using density plots of the estimated scores before and after matching to examine the overlap and distribution of scores between groups. Balance between matched groups was assessed using Absolute Standardized Mean Differences (ASMD), with an ASMD < 0.2 considered indicative of adequate covariate balance, based on comparisons of ASMD values before and after matching. Balance of predictors between the two groups was checked.

### Multivariate analysis

In multivariate analysis, a logistic regression model was performed to compare IMCU patients identified as “ICU-like” with a propensity-matched population from the ICU to identify patient factors related to the likelihood of being admitted to IMCU rather than ICU. Independent variables included age, sex, HFRS and CCI. The model was implemented using the finalfit package in R (Version 4.3.2), which facilitates the construction and presentation of regression models. Internally, finalfit employs a generalized linear model with binomial distribution and logit link function to estimate adjusted odd ratio (ORs) and their 95% confidence intervals (95%CIs). This method enables simultaneous adjustment for multiple covariates and ensures a transparent and reproducible workflow for statistical analysis. Statistical significance was set at *p* < 0.05.

### Ethics

Access to linked de-identified data in the HDD was performed in accordance with the French Reference Methodology procedure MR-005 for retrospective studies using HDD data, declaration signed by the teaching hospital of Tours (*MR005 number I4116221019*), as regulated by the French Data Protection Board *(Commission Nationale de l’Informatique et des Libertés, CNIL*). According to French data regulations, consent or information of each patient included was not required to use the French HDD de-identified data.

## Results

**Fig. 1:**
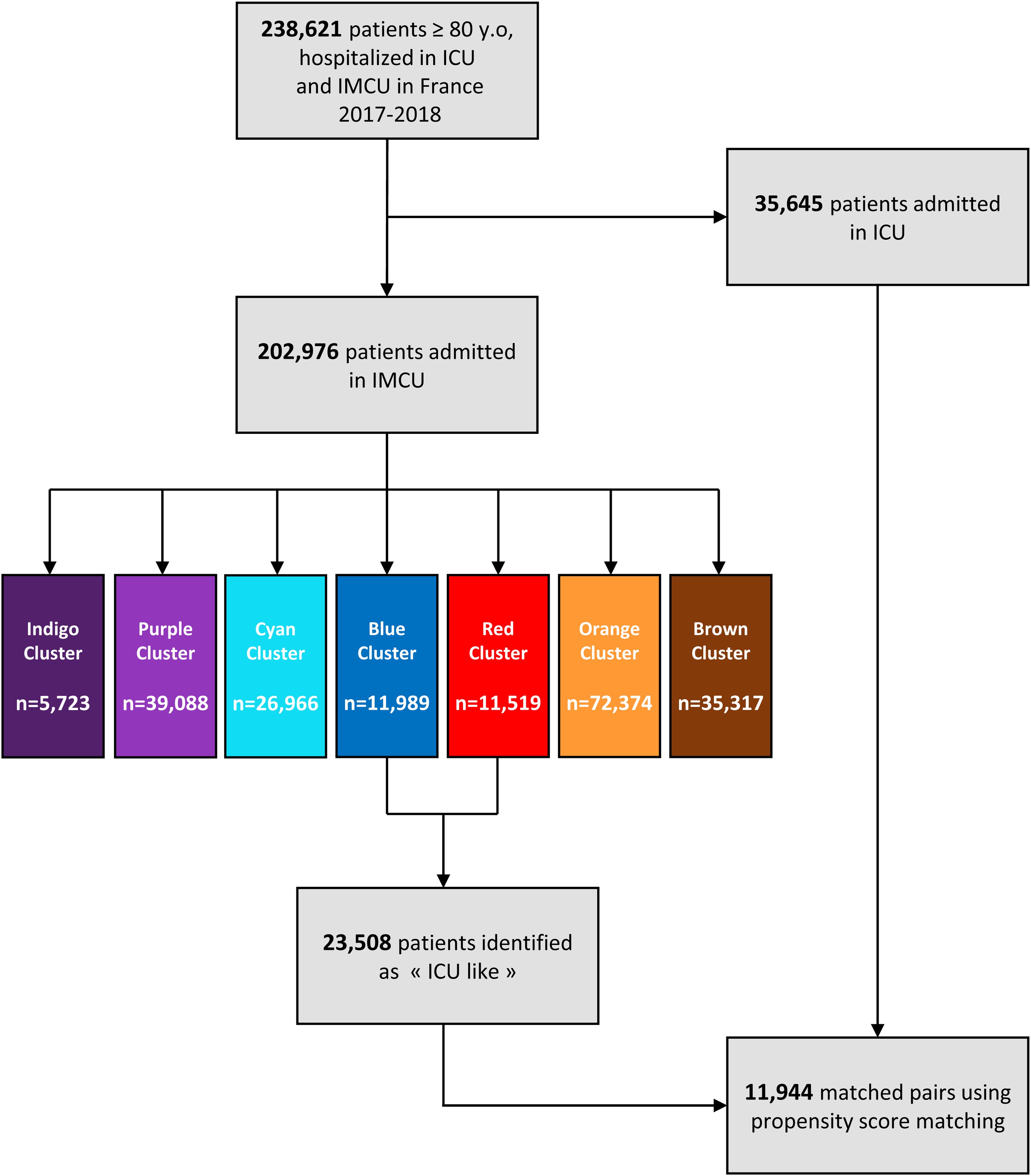
Flowchart of patient selection and clusters generated by latent class analysis. Intensive Care Unit (ICU); Intermediate Care Unit (IMCU).

A total of 238,621 very old patients were hospitalized in critical care units in France in 2017−18, including 202,976 to the IMCU and 35,645 to the ICU (Fig. 1).

**Fig. 2:**
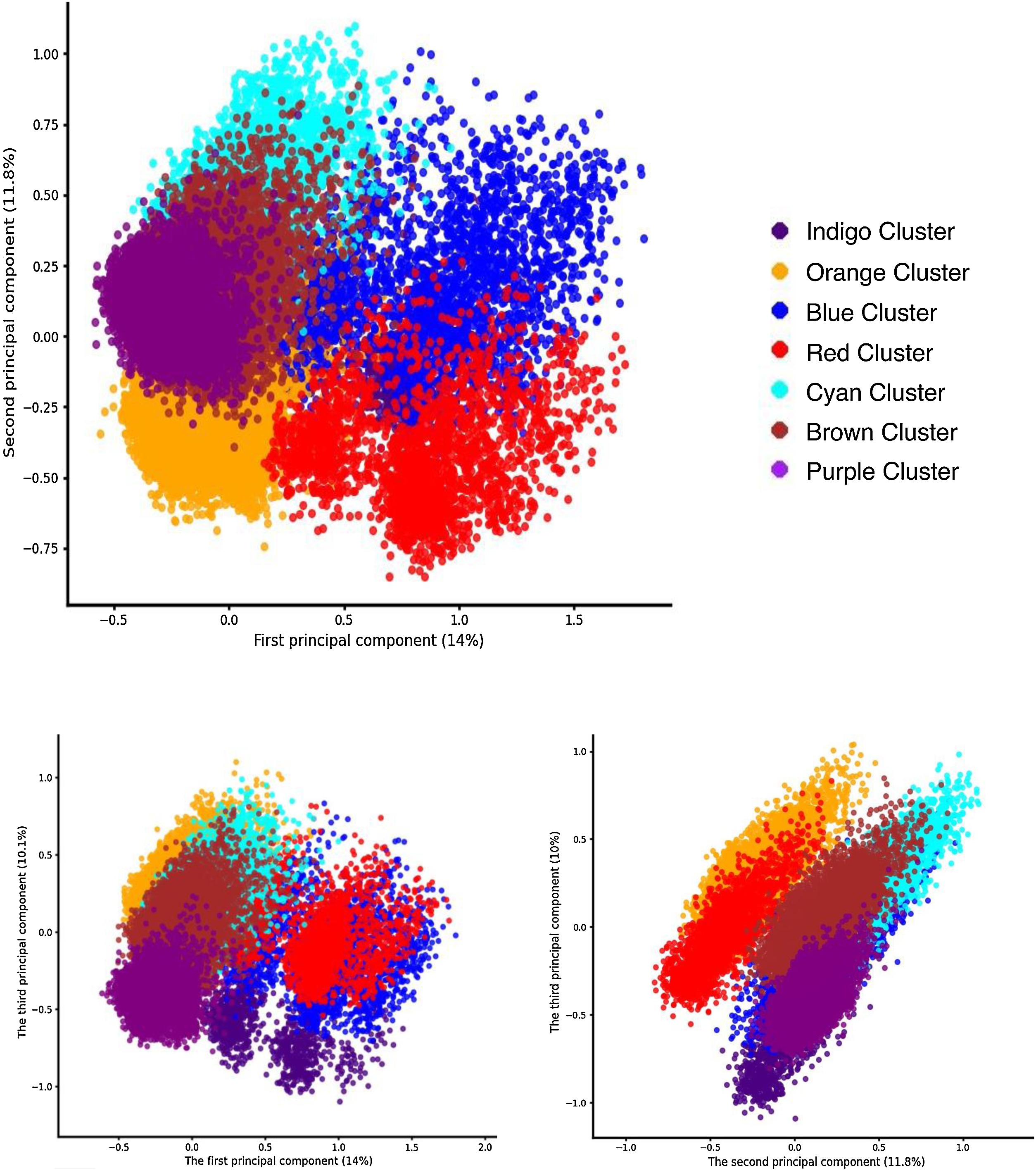
Graphical representation of latent class heterogeneity using a multiple correspondence analysis. Multiple correspondence analysis is performed to show graphically the heterogeneity of latent class. Red and Blue clusters were considered as "ICU-like".

The LCA results supported the hypothesis that distinct latent classes existed among IMCU patient, with a seven-class model selected as the optimal solution (Fig. 1, Supplemental Fig. S1). The seven-class model is graphically represented in Fig. 2 and the Supplemental Fig. S2.

The characteristics of very old patients hospitalized in the IMCU, grouped by latent classes, are provided in the Table 1, the Supplemental Table S3 and the Supplemental Fig. S2. The Orange, Cyan, Blue, and Red clusters were defined by the significant use of high-level care support but were clearly distinguishable based on mortality. The Orange and Cyan clusters had very low mortality rates whereas the Blue and Red clusters showed the highest mortality rates, with nearly two-thirds of patients dying during their IMCU stay. The Blue and Red clusters could therefore be considered "ICU-like" given their substantial use of high-level care support (26.3% and 17.5%, respectively) and high in-IMCU mortality (66.5% and 69%, respectively). Consistently, using MCA, we observed that the Blue and Red clusters were separated from all other clusters along the first principal component (Fig. 2). As the MCA is a low-dimensional visualization tool that captures a limited proportion of the total variance (here, the first two dimensions account for approximately 26% of inertia), distances and apparent separations in the MCA space may exaggerate within-class variability and should not be interpreted as evidence of additional latent classes. The apparent dispersion and visually bimodal pattern observed within the red and blue clusters in the MCA projection reflect the clinical heterogeneity of the ICU-like classes rather than the presence of an unmodeled latent substructure. The Blue cluster was mainly associated with respiratory organ dysfunction, often in combination with other diagnoses, whereas the Red cluster was primarily characterized by cardiac and circulatory failure. Sepsis affected more than 20% of the patients in these "ICU-like" clusters. Overall, very old "ICU-like" patients accounted for 11.6% of all very old patients hospitalized in IMCUs in France. Notably, the number of very old "ICU-like" patients admitted to IMCUs was 23,508 and the number of very old patients admitted to ICUs during the same period was 35,645.

**Table 1:** Baseline cluster profiles according to a seven-class model by latent class analysis (patients ≥ 80 y.o. hospitalized in IMCU, France, 2017–2018).

		Indigo	Purple	Cyan	Blue	Orange	Red	Brown	Overall
		*N* = 5723 (2.8%)	*N* = 39,088 (19.3%)	*N* = 26,966 (13.3%)	*N* = 11,989 (5.9%)	*N* = 72,374 (35.7%)	*N* = 11,519 (5.7%)	*N* = 35,317 (17.4%)	*N* = 202,976 (100%)
Age (Mean ± std)		87 ± 4.6	86 ± 4.2	86 ± 4.3	87 ± 4.5	86 ± 4.4	88 ± 4.5	86 ± 4.3	86.2 ± 4.4
Men^a^ (*n*, %)		2367 (41.4%)	15,612 (39.9%)	11,728 (43.5%)	5998 (50.0%)	31,415 (43.4%)	5462 (47.4%)	16,742 (47.4%)	89,324 (44%)
Charlson Comorbidity Index (Mean ± std)		1.3 ± 1.9	0.8 ± 1.6	1.4 ± 2.0	2.0 ± 2.5	1.3 ± 1.9	1.9 ± 2.2	1.5 ± 2.2	1.3 ± 2.0
Hospital Frailty Risk Score^a^ (Mean ± std)		5.3 ± 7.7	3.6 ± 6.5	4.9 ± 7.2	6.6 ± 8.1	4.1 ± 6.5	6.0 ± 7,9	5.0 ± 7.2	4.6 ± 7.0
Type of organ dysfunction (*n*, %)	Respiratory	0 (0%)	0 (0%)	26,966 (100%)	6490 (54.1%)	0 (0%)	0 (0%)	0 (0%)	33,456 (16%)
	Cardiac or circulatory	0 (0%)	0 (0%)	0 (0%)	0 (0%)	7374 (100%)	11,519 (100%)	0 (0%)	83,893 (41%)
	Neurological	5723 (100%)	39,088 (100%)	0 (0%)	0 (0%)	0 (0%)	0 (0%)	0 (0%)	44,811 (22%)
	Other	0 (0%)	0 (0%)	0 (0%)	5499 (45.9%)	0 (0%)	0 (0%)	35,317 (100%)	40,816 (20%)
Sepsis (*n*, %)		430 (7.5%)	1183 (3%)	2916 (10.8%)	2868 (23.9%)	4808 (6.6%)	2377 (20.6%)	4368 (12.4%)	18,950 (9%)
SAPSII > 40 (*n*, %)		830 (14.5%)	1013 (2.6%)	4333 (16.1%)	5651 (47.1%)	6061 (8.4%)	3428 (29.8%)	6741 (19.1%)	28,057 (13.8%)
High-level organ support (*n*, %)	Invasive ventilation	79 (1.4%)	44 (0.1%)	241 (0.9%)	469 (3.9%)	160 (0.2%)	258 (2.2%)	133 (0.4%)	1384 (1%)
	Non-invasive ventilation	117 (2.0%)	189 (0.5%)	4672 (17.3%)	2527 (21.1%)	3325 (4.6%)	1247 (10.8%)	895 (2.5%)	12,972 (6%)
	Inotropic and vasopressor support	50 (0.9%)	37 (0.1%)	330 (1.2%)	800 (6.7%)	870 (1.2%)	945 (8.2%)	525 (1.5%)	3557 (2%)
	Any of them	214 (3.7%)	247 (0.6%)	4930 (18.3%)	3154 (26.3%)	4082 (5.6%)	2017 (17.5%)	1436 (4.1%)	16,080 (8%)
Length of stay > 5 days (*n*, %)		3755 (65.6%)	29,854 (76.4%)	22,078 (81.9%)	7384 (61.6%)	55,502 (76.7%)	6370 (55.3%)	27,318 (77.4%)	152,261 (75%)
Death in IMCU (*n*, %)		2321 (40.6%)	0 (0%)	0 (0%)	7975 (66.5%)	3 (0%)	7948 (69%)	0 (0%)	18,247 (9%)

a Recorded but not included in the latent class analysis. Full categorical set of variables used to construct the latent classes, which remain available in Supplementary Table S3.

**Fig. 3:**
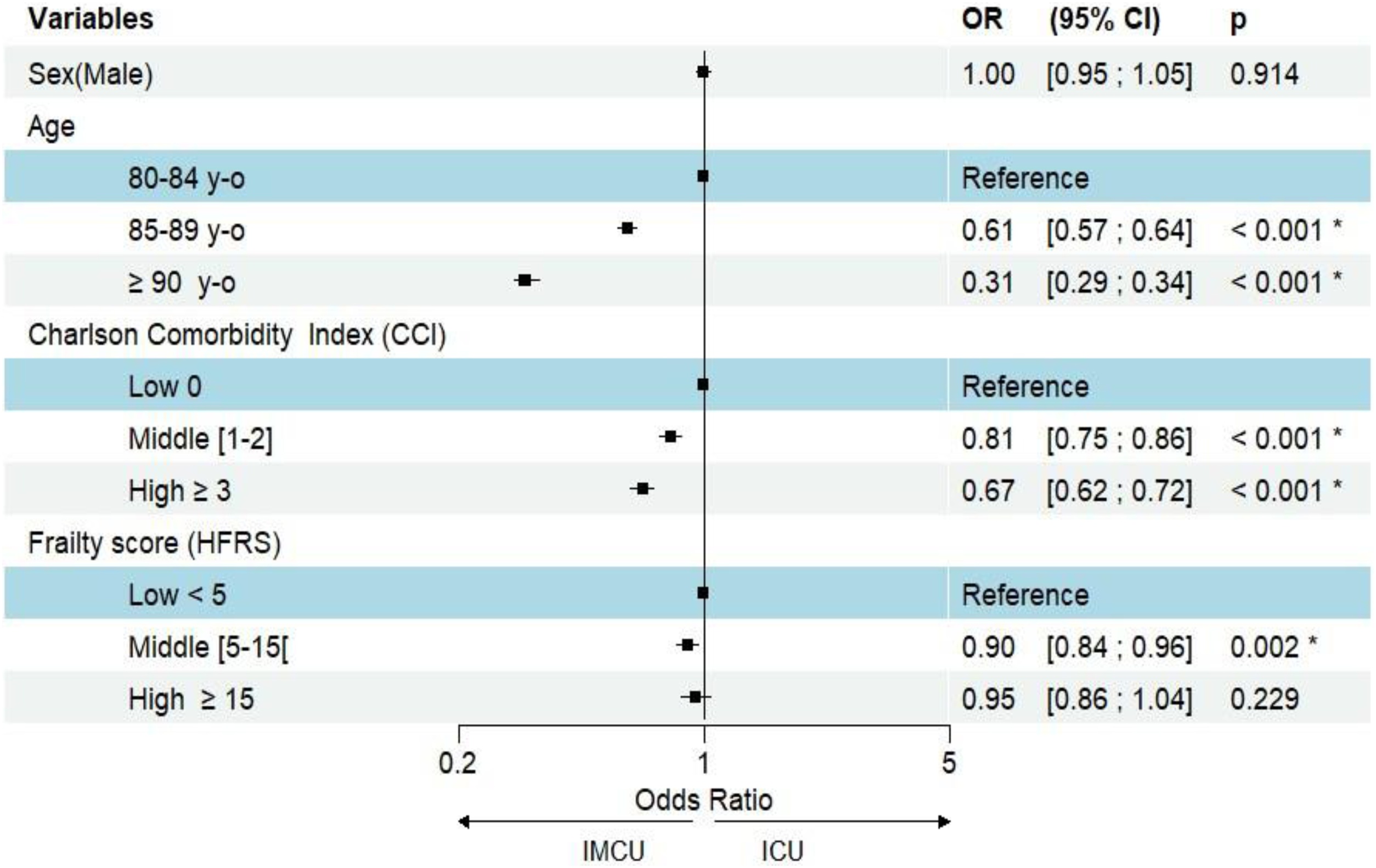
Forest plot of multivariate logistic regression analysis in propensity score-matched patient. The model assesses the effect of patient characteristics (age, sex, Hospital Frailty Risk Score, and Charlson Comorbidity Index) on the likelihood of admission to an Intermediate Care Unit (IMCU) rather than an Intensive Care Unit (ICU) within the propensity score-matched population. Propensity score was estimated based on similar use of high-level organ support, SAPS II distribution, and type of organ dysfunction (determined by primary diagnosis in the ICU or IMCU). Matching was performed using the “k-nearest neighbor” method. Results are presented adjusted odds ratios (OR) and 95% confidence intervals (95% CI) for characteristics associated with admission to the ICU or IMCU.

Of the 23,508 patients classified as “ICU-like”, 11,944 were matched by propensity score to ICU patients with similar use of high-levels organ support, similar SAPS II distribution, and corresponding types of organ dysfunction based on the primary diagnosis in the ICU or IMCU (Fig. 1). The baseline characteristics of ICU and “ICU-like” populations as well as those of the matched cohort are presented in Table 2 and the Supplemental Figure S3. After propensity score matching, the multivariate logistic regression identified age as the strongest determinant to IMCU admission, with very old patients being significantly more likely to be admitted to the IMCU over the ICU (Fig. 3). Comorbidity status, as quantified by the Charlson Comorbidity Index was also an independent predictor for admission to IMCU over ICU. Conversely, frailty exhibited only a marginal effect: a moderate Hospital Frailty Risk Score (HFRS 5-14) was significantly associated with admission (OR = 0.90, 95% CI [0.84, 0.96]), but a high level (HFRS > 15) was not (OR = 0.95, 95% CI [0.86, 1.04]). Finally, sex showed no detectable influence (OR = 1.00, 95% CI [0.95, 1.05])

**Table 2:** Characteristic of patients ≥ 80 y.o hospitalized in “ICU-like” and ICU (France, 2017-2018), before and after propensity score matching.

Characteristics (*n*, %)		Before matching		After matching	
		“ICU like” population (*N* = 23,508)	ICU population (*N* = 35,645)	Matched "ICU like" population (*N* = 11,944)	Matched ICU population (*N* = 11,944)
Men		11,460 (49%)	18,840 (53%)	6019 (50%)	5838 (49%)
Age	[80−84] y.o.	7263 (31%)	19,259 (54%)	3957 (33%)	6006 (50%)
	[85−89] y.o.	8969 (38%)	12,390 (35%)	4618 (39%)	4281 (36%)
	≥90 y.o.	7276 (31%)	3996 (11%)	3369 (28%)	1657 (14%)
Charlson Comorbidity Index	CCI 0	9814 (42%)	17,351 (49%)	4890 (41%)	5782 (48%)
	CCI [1,2]	6109 (26%)	9220 (26%)	3166 (27%)	3025 (25%)
	CCI ≥ 3	7585 (32%)	9074 (25%)	3888 (33%)	3137 (26%)
Hospital Frailty Risk Score	HFRS < 5	13,612(58%)	22,718 (64%)	6,720 (56%)	7,562 (63%)
	HFRS [5–14]	6654(28%)	9241 (26%)	3514 (29%)	2995 (25%)
	HFRS ≥ 15	3242(14%)	3686 (10%)	1710 (14%)	1387 (12%)
Type of organ dysfunction	Respiratory	6490 (28%)	13,077 (37%)	3608 (30%)	3482 (29%)
	Cardiac or circulatory	11,519 (49%)	9215 (26%)	3203 (27%)	3398 (28%)
	Neurological	0 (0%)	4199 (12%)	0 (0%)	0 (0%)
	Other	5499 (23%)	9154 (26%)	5133 (43%)	5064 (42%)
Sepsis		5245 (22 %)	11,667 (33 %)	3057 (26 %)	3127 (26 %)
SAPSII	SAPSII < 40	14,429 (61 %)	9081 (27 %)	5559 (47 %)	5529 (46 %)
	SAPSII [40–49]	4094 (17 %)	7547 (22 %)	2864 (24 %)	2799 (23 %)
	SAPSII ≥ 50	4985 (21 %)	17,502 (51 %)	3521 (29 %)	3616 (30 %)
High-level organ support	Invasive ventilation	727 (3 %)	15,444 (43 %)	726 (6 %)	653 (5 %)
	Non-invasive ventilation	3774 (16 %)	3933 (11 %)	3656 (31 %)	3657 (31 %)
	Inotropic and vasopressor support	1745 (7 %)	15,366 (43 %)	1724 (14 %)	2052 (17 %)
Death in the critical care unit		15,923 (68 %)	12,313 (35 %)	8324 (70 %)	2084 (17 %)

Propensity score was estimated based on similar use of high-level organ support, SAPS II distribution, and type of organ dysfunction (determined by primary diagnosis in the ICU or IMCU). Matching was performed using the “k-nearest neighbor” method.

## Discussion

We conducted a nationwide study analyzing all patients aged 80 years or older who were hospitalized in IMCUs in France over a two-year period. Our findings revealed significant heterogeneity within this population. Notably, we identified a distinct “ICU-like” subgroup of very old patients in IMCUs, characterized by frequent advanced organ support and high mortality rates. During the study period, 23,508 very old patients with an “ICU-like” profile were admitted to IMCUs, compared to 35,645 admitted to ICUs. Patients in the “ICU-like” subgroup showed a fourfold increase in mortality risk compared to matched ICU patients. Age emerged as the primary factor influencing triage decisions between IMCU and ICU admission.

Interest in IMCUs has increased in both Europe and United States; however, comparisons are complicated due to differences in definitions [23]. In France, decrees and circulars issued in 2002 defined the scope of activities of ICU in a set of 250 recommendations [24]. The same decrees also defined IMCU but their regulatory specifications were much more limited. In 2022, a division of the French Ministry of Health provided a new decree to clarity the organization of IMCUs [25]. IMCUs are now classified as polyvalent or specialized. Polyvalent IMCUs provide care, within a dedicated unit, for patients who are at risk of developing one or more acute failures that directly threaten their vital or functional prognosis. Specialized IMCUs (such as cardiology, neurovascular, and hematology) manage patients who have or are at risk of developing acute failures related to the pathology of the organ associated with their specialty. IMCUs have a level of nursing staff (and costs) lower than ICU although higher than in the general wards and can manage patients that may require temporary life-support methods, pending, if necessary, a transfer to the ICU. In this study, we selected very old IMCU patients who were not admitted to ICU and further demonstrated that more than 10% of these patients were “ICU-like”. The question of why these patients were not hospitalized in ICU is unclear. Admission to an IMCU may optimize hospital resource allocation by reducing unnecessary ICU admissions and though improve patient safety by avoiding inappropriate placement in conventional wards [18]. The very old “ICU-like” patients did not align with this approach: although they received advanced organ support (assuming that the patient were Full Code), none were transferred to the ICU despite a high mortality rate. In contrast, the indigo cluster generated by the LCA included patients with high probability of death but who did not receive high-level organ support, reflecting a clear palliative care approach. We may hypothesize that the “ICU-like” cluster represented patients with a time-limited trial of intensive care treatment and a subsequent withdraw of organ support due to absence of improvement. Time-limited trial is defined as “an agreement between clinicians and a patient/family to use certain medical therapies over a defined period to see if the patient improves or deteriorates according to agreed-on clinical outcomes” [19]. Time-limited trials are usually conducted in ICUs [20], but the IMCU setting may have been preferred regarding this very old population. This choice could reflect an effort to minimize patient discomfort by reducing alarm-related stress and fostering a calmer environment while accepting a less intensive level of care than in the ICU. Finally, we observed that patients in the “ICU-like” subgroup had a fourfold higher risk of mortality compared with matched ICU patients. However, it is difficult to determine whether this finding implies that very old patients admitted to the IMCU despite meeting criteria for ICU admission would benefit from ICU admission, as causality cannot be established from this observation. We used causal inference tools (propensity score methods) to suggest an association, but we cannot formally demonstrate a causal effect. Given that observational studies are sometimes overinterpreted, we chose to emphasize the observed findings rather than untested causal assumptions. Overall, there is a timely effort for addressing the question of appropriateness of admission in IMCUs in France [3,18]. This issue is particularly critical for very old patients, who account for 30% of all IMCU admissions [7] and include nearly 10,000 patients annually with an “ICU-like” profile.

Understanding the decision-making process that results in admission to an IMCU instead of an ICU is particularly complex. Using latent class analysis, we identified distinct IMCU patient clusters, each characterized by unique clinical features and resource use patterns. In this study, age was the major determinant driving admission to the IMCU over the ICU. The burden of chronic diseases (assessed by the CCI) and the frailty status (assessed by the HFRS) were used in a lesser extent. Relying solely on age as a categorical criterion risks oversimplifying critical care decisions [21]. Although this approach may streamline patient selection, it fails to capture the diversity of the aging population or the wide variability in physiological resilience among older adults. Such rigid criteria may inappropriately exclude older patients with adequate physiological reserve and rehabilitation potential to derive meaningful benefit from ICU care. Importantly, the ESICM consensus-based recommendations for the management of very old patients in intensive care stated that chronological age should not be used alone as a criterion for admission to the ICU (strong recommendation) [8]. In our study, the frailty status was poorly associated with the triage decisions, despite recommendations to assess health status prior to hospital admission [22,23].

The results of this study should be interpreted considering the methodological choices that we made. First, the definition of very old patient is variable in the literature and vary from 60 years (as defined by the World Health Organization) to 90 years [24]. We chose 80 years as the threshold for defining very old patients because the increase of mortality in ICU is greater after this age [2,25,26] and frailty is elevated in this population [23,27]. Finally, 80 years is the most widely accepted cutoff for defining a very old patient in the literature in ICU [23]. Second, surgical patients were not included in this study because the prognosis for planned perioperative situations is already well-documented and differs significantly from acute organ dysfunction in very old patients [28]. Third, we selected the 2017–2018 period because the COVID-19 pandemic disrupted usual conditions and post-pandemic care pathways had not yet returned to their pre-pandemic norms [7]. We previously demonstrated that the 2017–2018 period was a representative period for trends in IMCU and ICU admissions in France [7].

Some limits of the study should be underscored and discussed. First, it is unknown, using this ICD-10 coding data, whether the patients hospitalized in the IMCU had "do not resuscitate" orders and/or were denied ICU admission, this information not being reported in the hospital discharge coded resume. Similarly, deaths in IMCU preceded by a decision to withhold or withdraw life sustaining treatment could not be identified. Because this information is unavailable, the analysis may indirectly attempt to capture the decision not to escalate care (i.e., not transferring patients to the ICU). This limitation stems from the nature of the database, but it has major implications for interpretation. Next, the frailty status can be examined via multiple tools [22]. In the clinical practice, the Clinical Frailty Scale (CFS) is recommended to be included in the decision process [23] as it has proven reliability and is consistently associated with both hospital and long-term survival [29,30]. As the CFS is not reported in the coded resume, we used the HFRS, which has been validated as a surrogate marker for the frailty status in older people [14], demonstrating its utility in predicting adverse outcomes in health systems [10,13,31]. Moreover, we have previously demonstrated that frailty status, assessed using the Hospital Frailty Risk Score, was associated with outcomes in this type of database [ref EER, ref MDM]. Finally, French hospital administrative databases are powerful tools for capturing global trends but are limited for granular analyses. We therefore adopted a categorization aimed at identifying the most likely predominant organ dysfunction, because a more detailed classification would have substantially increased model complexity, carried a high risk of misclassification, and reduced the stability and interpretability of the LCA, without a clear gain in clinical relevance. Our results constitute an important first step in demonstrating the importance of ICU-like patients hospitalized in IMCU, but with this methodology we are limited in our ability to fully explain their heterogeneity. Future studies should incorporate more granular clinical data to further characterize and interpret these findings. Overall, the strengths and limitations of using HDD for epidemiological purposes have already been extensively discussed [2]. Eventually, we studied the whole French population; our results could hardly be extrapolated to countries with different healthcare systems or IMCU/ICU admission policy.

In conclusion, real world data from the entire French population hospitalized in IMCUs were analyzed and a substantial number of very old patients were admitted to IMCU despite meeting criteria for ICU admission. Given the ongoing demographic shift toward an older population, there is an urgent need to clarify the admission criteria and objectives of IMCU stays for older critically ill patients.

## CRediT authorship contribution statement

AM, LGG and AG conceived and designed the study and were involved in drafting the manuscript. LG performed the data retrieval. AKS and LG performed the statistical analysis. AM, AKS, LG, EL, LGG and AG were involved in the interpretation of the data, in drafting the manuscript and made critical revisions to the discussion section. All authors read and approved the final version to be published.

## Ethics approval and consent to participate

No nominative, sensitive or personal data of patients have been collected. Our study involved the reuse of already recorded and anonymized data. The study falls within the scope of the French Reference Methodology MR-005 (declaration 2205437 v0, august 22nd, 2018, by the Teaching Hospital of Tours), which require neither information nor consent of the included individuals. This study was consequently registered with the French Data Protection Board (*CNIL* MR-005 number I4116221019).

## Consent for publication

Not applicable.

## Funding

This study was supported by a grant from 10.13039/501100001677Inserm and the French Ministry of Health in the context of MESSIDORE call operated by IReSP (GENIALLY, 2022, Inserm-MESSIDORE N° 72).

## Availability of data and material

Restrictions apply to the availability of these data and so are not publicly available. However, aggregated data are available from the authors upon reasonable request and with the permission of the institution, or via the national Hub ATIH (https://www.atih.sante.fr/)

## Declaration of competing interest

The authors declare the following financial interests/personal relationships which may be considered as potential competing interests:

GUILLON Antoine reports financial support was provided by INSERM. KASSA-SOMBO Arthue reports financial support was provided by INSERM. Leslie GRAMMATICO-GUILLON reports financial support was provided by INSERM. If there are other authors, they declare that they have no known competing financial interests or personal relationships that could have appeared to influence the work reported in this paper.
