# Supplementary material for: Characterizing ICU-like profiles of very old patients hospitalized in medical intermediate care units in France: A clustering analysis of a nationwide population-based study
Source: Ann Intensive Care. 2026 Jan 30;16:100031. doi: 10.1016/j.aicoj.2026.100031 (PMC12934429; doi:10.1016/j.aicoj.2026.100031)
Supplement: Supplementary file 1 [file mmc1.docx]

**Supplementary materials: Tables**

**Supplemental Table S1.** Organ classification of ICD-10 primary diagnosis.

**Supplemental Table S2.** French CPT codes of high-level organ support

**Supplemental Table S3.** Cross tabulation characteristics within each latent class.

**Supplemental Table S1.** Organ classification of ICD-10 primary diagnosis.

| **ICD-10 classification by organ** | **List of ICD-10 codes** |
| --- | --- |
| CARDIAC OR CIRCULATORY | E86, E86, I00-I25, I300, I301, I30-I31, I328, I34-I37, I401-I409, I418-I42, I430, I431-I519, I70-I721, I723-I729, I74, I77, I790-I791, I792-I80, I821-I822, I828-I829, I95, I970-I971, I978-I979, L97, R00-R01, R03, R55, R570-R571, R578, R579, R58, T78, T811 |
| *including septic* | A021, A327-A329, A392-A395, A40, A41, A427, A440, A481-A484, A483, A75-A79, A90-A99, B332, B334, B377, B464, B49, B570, B572, I320-I321, I33, I38-I400, I410-I412, I52, I980-I981, J853, J853, R572 |
| RESPIRATORY | E840, J30-J31, J33, J341-J35, J38, J40-J439, J441-J47, J60-J70, J80, J81-J84, J92-J95, J960, J961-J99, R04-R091, R092, U04 |
| *including septic* | A15-A16, A19, A310, A36-A38, A420, A430, A481, A691, A70, B012, B052, B250, B342, B371, B44, B450, B460, B510, B55, B573, B583, B59, I26-28, J00-J06, J09-J18, J20-J22, J32, J340, J36-J37, J39, J440, J850-J852, J86, J90-J91, R093-R094 |
| NEUROLOGICAL | E100, E140, E15, G08-G09, I60-I680, I682-I69, R20, R25-R29, R40-R49, R51-R52, R56, R90 |
| *including septic* | A17, A321, A390, A80-A89, B003-B004, B010-B011, B020-B023, B050-B021, B261-B262, B375-B376, B451, B461, B500, B56, B571, B574, B582, B690, G00-G07, I681 |
| Hematologic-oncologic | C00-D97, R91-R92 |
| OTHERS | R96, R99, E841, I81, I820, I85, I982, K20-K31, K35-K38, K40-K46, K50-K52, K55, K56-K63, K66, K70-K76, K80, K85-K86, K90-K93, R10-R19, R74, N30, N33-N34, N390, N41, N45, N51, N61, N70-N77, D80-D89, E102, E112, E132, E142, E83-E85, E87, I722, I73, I78, I823, L50-L54, L93-L95, M05-M14, M30-M36, M359, M60-M61, M63, N00-N079, N081-N087, N089, R50, R79, B20-B24, B520, I78, N080, N088, T96-T97, E00-E059, E061-E07, E101, E111, E121, E131, E141, E20-E35, E40-E64, E70-E72, E74-E80, E88-E89, R63-R64, L10-L14, M15-M25, M40-M54, M62, M65-M79, M80-M99, R02, R21-R22, S00-T35, T79, T90-T95, T98, F00-F99, R78, T51-T57, X60-X94, X85-Y09, T36-T50, T80-T801, T803-T813, T815-T825, T828-T834, T837-T844, T848-T856, T858-T873, T875-T879, T881-T88, Y40-Y84 |
| *including septic* | A00-A020, A022-A09, A421, B007-B009, B15-B19, B251-B259, B263, B27, B462, B581, B65-B689, B691-B83, D73, K65, K67, K77, K81-K83, K87, A18, A20-A28, A30, A311-A319, A32, A398-A399, A428-A429, A438-A439, A448-A449, A488, A49, B005, B018-B019, B027-B029, B03, B04, B053-B059, B09, B268, B551-B559, B575, B588-B589, B95-B97, B508-B509, B518-B519, B528-B529, B53-B54, B99, N31-N32, N35-N389, N391-N399, N60-N609, N611-N64, N92-N93, R30-R39, A391, E060, E16, A422, A431, A441, A46, A480, B000-002, L00-L08, M00-M03, T802, T814, T826-T827, T835-T836, T845-T847, T857, T874, T880 |

**Supplemental Table S2.** French CPT codes of high-level organ support

| **CPT procedure** | **Codes** | **Labels** |
| --- | --- | --- |
| Inotropic and vasopressor support | EQLF001 | Continuous intravenous injection of dobutamine or dopamine at a rate of less than 8 micrograms per kilogram per minute [µg/kg/min], or dopexamine outside the neonatal period, per 24 hours |
|  | EQLF003 | Continuous intravenous injection of dobutamine or dopamine at a rate exceeding 8 micrograms per kilogram per minute [µg/kg/min], adrenaline or noradrenaline outside the neonatal period, per 24 hours |
| Non-Invasive mechanical ventilation, Continuous Positive Airway Pressure (CPAP) | GLLD003 | Spontaneous ventilation by face mask, nasal cannula or nasopharyngeal tube, without inspiratory support, with positive expiratory pressure [VS-PEP] [Continuous positive airway pressure] [CPAP], per 24 hours |
| Non-Invasive mechanical ventilation, Bilevel Positive Pressure (BPAP) | GLLD019 | Non-invasive barometric or volumetric ventilation by face mask for at least 2 hours cumulative over a 12-hour period, for acute respiratory failure |
|  | GLLD012 | Continuous mechanical ventilation with face mask for ventilatory support, per 24 hours |
| Invasive mechanical ventilation | GLLD015 | Intratracheal mechanical ventilation with positive expiratory pressure [PEEP] less than or equal to 6 and FiO2 less than or equal to 60%, per 24 hours |
|  | GLLD008 | Intratracheal mechanical ventilation with positive expiratory pressure [PEEP] greater than 6 and/or FiO2 greater than 60%, per 24 hours |
|  | GLLD004 | Intratracheal mechanical ventilation with positive expiratory pressure [PEEP] greater than 6 and/or FiO2 greater than 60%, with alternate prone positions every 24 hours |

**Supplemental Table S3.** Full cross tabulation characteristics within each latent class

|  |  |  |  |  |  |  |  |  |  |  |  |
| --- | --- | --- | --- | --- | --- | --- | --- | --- | --- | --- | --- |
|  |  |  |  | **Indigo** | **Purple** | **Cyan** | **Blue** | **Orange** | **Red** | **Brown** |  |
|  | **Characteristics (n,%)** |  |  | N = 5,723 (2.8%) | N = 39,088 (19.3%) | N = 26,966 (13.3%) | N = 11,989 (5.9%) | N = 72,374 (35.7%) | N = 11,519 (5.7%) | N = 35,317 (17.4%) |  |
|  |  |  |  |  |  |  |  |  |  |  |  |
|  | **Age** | *[80-84] y.o.* |  | 1,888 (33.0%) | 17,623 (45.1%) | 11,622 (43.1%) | 3,951 (33.0%) | 28,265 (39.0%) | 3,312 (28.9%) | 15,474 (43.8%) |  |
|  |  | *[85-89] y.o.* |  | 2,153 (37.6%) | 14,240 (36.4%) | 9,815 (36.4%) | 4,543 (37.9%) | 27,050 (37.4%) | 4,426 (38.4%) | 12,890 (36.5%) |  |
|  |  | *≥ 90* |  | 1,682 (29.4%) | 7,225 (18.5%) | 5,529 (20.5%) | 3,495 (29.2%) | 17,059 (23.6%) | 3,781 (32.8%) | 6,953 (19.7%) |  |
|  | **Charlson Comorbidity Index** | *CCI = 0* |  | 3,345 (58.5%) | 27,740 (71.0%) | 14,064 (52.2%) | 4,974 (41.5%) | 40,256 (55.6%) | 4,840 (42.0%) | 18,585 (52.6%) |  |
|  |  | *CCI = [1-2]* |  | 1,302 (22.8%) | 7,267 (18.6%) | 6,655 (24.7%) | 3,177 (26.5%) | 16,840 (23.3%) | 2,932 (25.5%) | 8,993 (25.5%) |  |
|  |  | *CCI ≥ 3* |  | 1,076 (18.8%) | 4,081 (10.4%) | 6,247 (23.2%) | 3,838 (32%) | 15,278 (21.1%) | 3,747 (32.5%) | 7,739 (21.9%) |  |
|  | **Type of organ dysfunction** *(determined by primary diagnosis in the IMCU)* | *Respiratory* |  | 0 (0%) | 0 (0%) | 2,6966 (100%) | 6,490 (54.1%) | 0 (0%) | 0 (0%) | 0 (0%) |  |
|  |  | *Cardiac or circulatory* |  | 0 (0%) | 0 (0%) | 0 (0%) | 0 (0%) | 7,374 (100%) | 11,519 (100%) | 0 (0%) |  |
|  |  | *Neurological* |  | 5,723 (100%) | 39,088 (100%) | 0 (0%) | 0 (0%) | 0 (0%) | 0 (0%) | 0 (0%) |  |
|  |  | *Hematologic-oncologic* |  | 0 (0%) | 0 (0%) | 0 (0%) | 1293 (10.8%) | 0 (0%) | 0 (0%) | 5832 (16.5%) |  |
|  |  | *Musculoskeletal* |  | 0 (0%) | 0 (0%) | 0 (0%) | 1066 (8.9%) | 0 (0%) | 0 (0%) | 5700 (16.1%) |  |
|  |  | *Nephro-immunological* |  | 0 (0%) | 0 (0%) | 0 (0%) | 764 (6.4%) | 0 (0%) | 0 (0%) | 4286 (12.1%) |  |
|  |  | *Other* |  | 0 (0%) | 0 (0%) | 0 (0%) | 2376 (18.8%) | 0 (0%) | 0 (0%) | 15,818 (44,8%) |  |
|  | **Sepsis** |  |  | 430 (7.5%) | 1,183 (3%) | 2,916 (10.8%) | 2,868 (23.9%) | 4,808 (6.6%) | 2,377 (20.6%) | 4,368 (12.4%) |  |
|  | **SAPSII** > 40 | *< 40* |  | 4893 (85.5%) | 38075 (97.4%) | 22633 (83.9%) | 6338 (52.9%) | 66313 (91.6%) | 8091 (70.2%) | 28576 (80.9%) |  |
|  |  | *[40-49]* |  | 291 (5.1%) | 640 (1.6%) | 3279 (12.2%) | 2676 (22.3%) | 4674 (6.5%) | 1418 (12.3%) | 5037 (14.3%) |  |
|  |  | *≥ 50* |  | 539 (9.4%) | 373 (1%) | 1054 (3.9%) | 2975 (24.8%) | 1387 (1.9%) | 2010 (17.4%) | 1704 (4.8%) |  |
|  | **High-level organ support** | *Invasive ventilation* |  | 79 (1.4%) | 44 (0.1%) | 241 (0.9%) | 469 (3.9%) | 160 (0.2%) | 258 (2.2%) | 133 (0.4%) |  |
|  |  | *Non-invasive ventilation* |  | 117 (2.0%) | 189 (0.5%) | 4,672 (17.3%) | 2,527 (21.1%) | 3,325 (4.6%) | 1,247 (10.8%) | 895 (2,5%) |  |
|  |  | *CPAP* |  | 26 (0.5%) | 72 (0.2%) | 1,297 (4.8%) | 741 (6.2%) | 1,116 (1.5%) | 362 (3.1%) | 338 (1%) |  |
|  |  | *BPAP* |  | 93 (1.6%) | 123 (0.3%) | 3,695 (13.7%) | 1,972 (16.4%) | 2,350 (3.2%) | 934 (8.1%) | 588 (1.7%) |  |
|  |  | *Vasopressor* |  | 50 (0.9%) | 37 (0.1%) | 330 (1.2%) | 800 (6.7%) | 870 (1.2%) | 945 (8.2%) | 525 (1.5%) |  |
|  |  | *Any of them* |  | 214 (3.7%) | 247 (0.6%) | 4,930 (18.3%) | 3,154 (26.3%) | 4,082 (5.6%) | 2,017 (17.5%) | 1,436 (4.1%) |  |
|  | **Length of stay > 5 days** |  |  | 3755 (65.6%) | 29854 (76.4%) | 22078 (81.9%) | 7384 (61.6%) | 55502 (76.7%) | 6370 (55.3%) | 27318 (77.4%) |  |
|  | **Death** | *In IMCU* |  | 2,321 (40.6%) | 0 (0%) | 0 (0%) | 7,975 (66.5%) | 3 (0%) | 7,948 (69%) | 0 (0%) |  |
|  |  | *In hospital stay* |  | 5723 (100%) | 0 (0%) | 0 (0%) | 11989 (100%) | 0 (0%) | 11519 (100%) | 0 (0%) |  |
|  |  | *At one year* |  | 5723 (100%) | 7022 (18%) | 7798 (28.9%) | 11989 (100%) | 21063 (29.1%) | 11519 (100%) | 9908 (28.1%) |  |
|  |  |  |  |  |  |  |  |  |  |  |  |
