# Supplementary material for: Characterizing ICU-like profiles of very old patients hospitalized in medical intermediate care units in France: A clustering analysis of a nationwide population-based study
Source: Ann Intensive Care. 2026 Jan 30;16:100031. doi: 10.1016/j.aicoj.2026.100031 (PMC12934429; doi:10.1016/j.aicoj.2026.100031)
Supplement: Supplementary file 2 [file mmc2.pptx]

## Slide 1
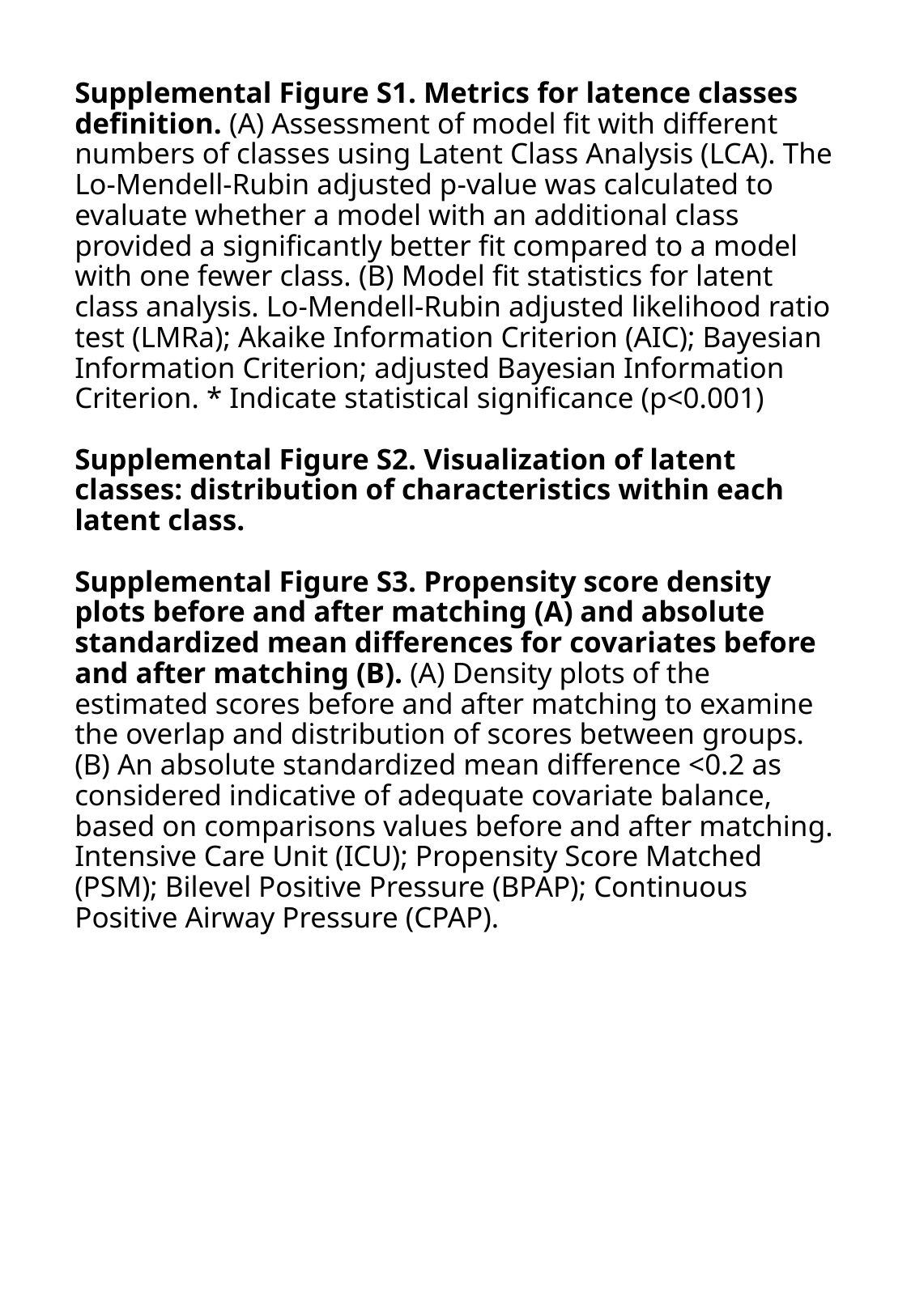

# Supplemental Figure S1. Metrics for latence classes definition. (A) Assessment of model fit with different numbers of classes using Latent Class Analysis (LCA). The Lo-Mendell-Rubin adjusted p-value was calculated to evaluate whether a model with an additional class provided a significantly better fit compared to a model with one fewer class. (B) Model fit statistics for latent class analysis. Lo-Mendell-Rubin adjusted likelihood ratio test (LMRa); Akaike Information Criterion (AIC); Bayesian Information Criterion; adjusted Bayesian Information Criterion. * Indicate statistical significance (p<0.001)   Supplemental Figure S2. Visualization of latent classes: distribution of characteristics within each latent class.   Supplemental Figure S3. Propensity score density plots before and after matching (A) and absolute standardized mean differences for covariates before and after matching (B). (A) Density plots of the estimated scores before and after matching to examine the overlap and distribution of scores between groups. (B) An absolute standardized mean difference <0.2 as considered indicative of adequate covariate balance, based on comparisons values before and after matching. Intensive Care Unit (ICU); Propensity Score Matched (PSM); Bilevel Positive Pressure (BPAP); Continuous Positive Airway Pressure (CPAP).

## Slide 2
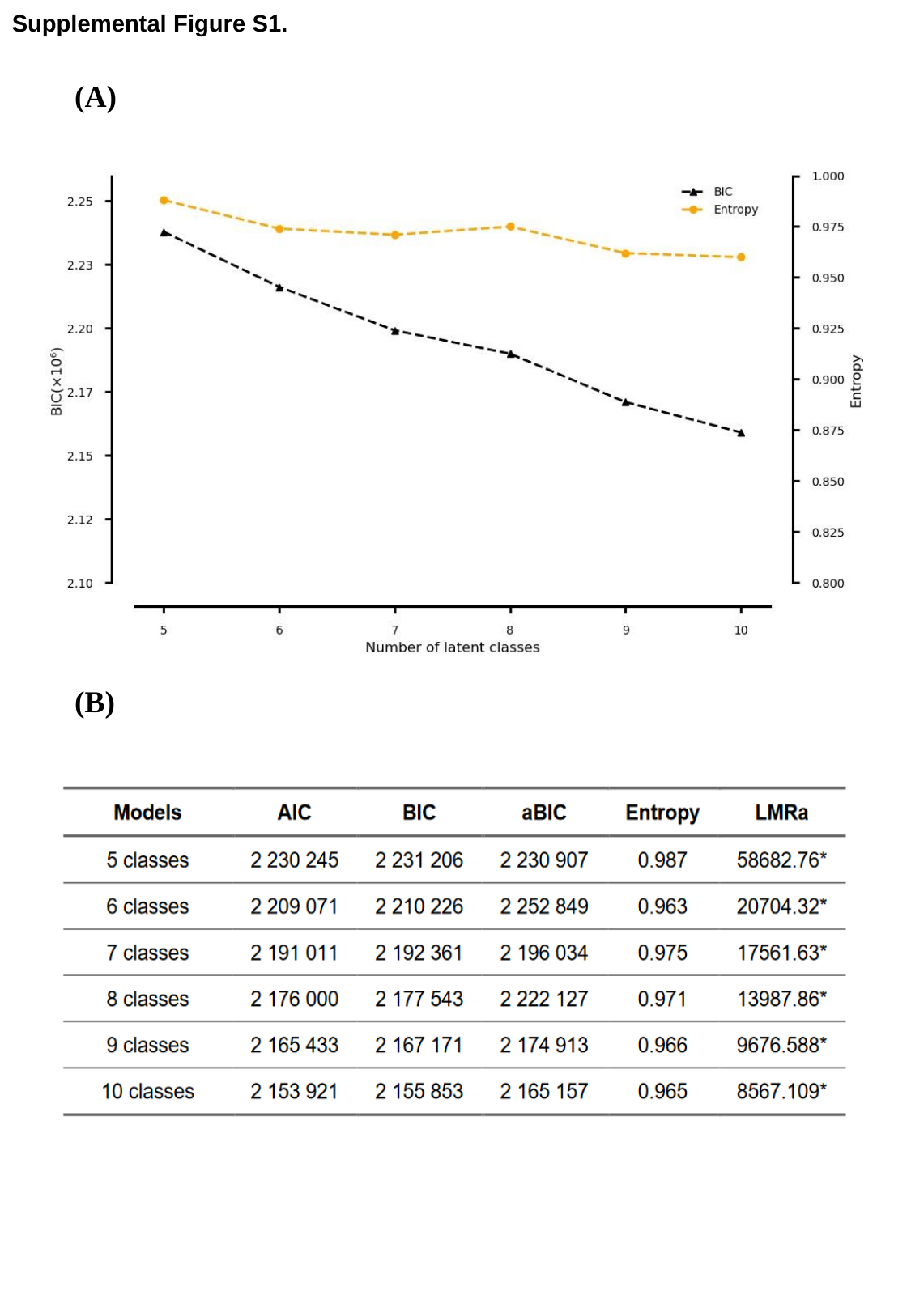

Supplemental Figure S1.
(A)
(B)

## Slide 3
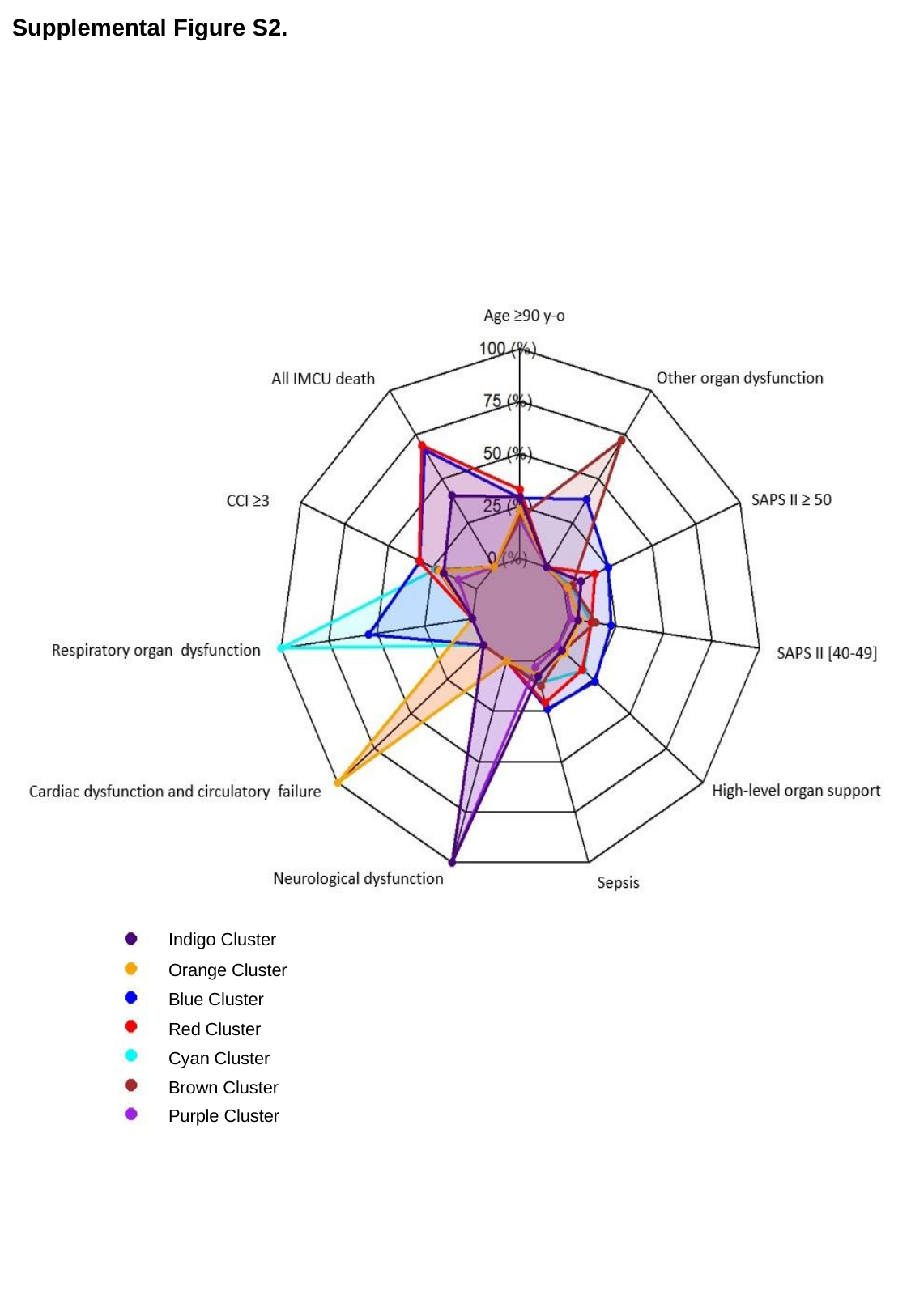

Supplemental Figure S2.
Indigo Cluster
Orange Cluster
Blue Cluster
Red Cluster
Cyan Cluster
Brown Cluster
Purple Cluster

## Slide 4
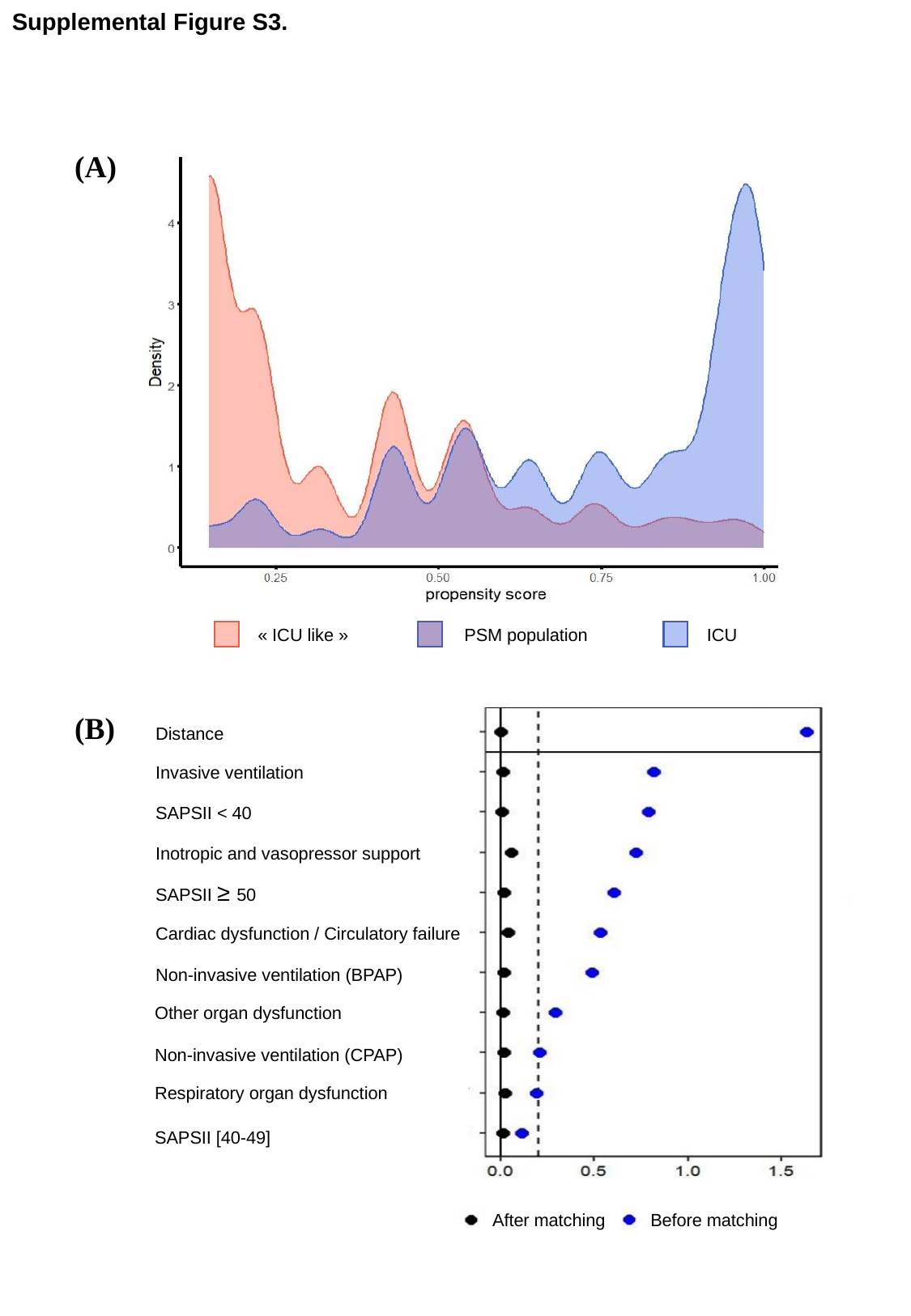

Supplemental Figure S3.
(A)
« ICU like »
PSM population
ICU
Distance
Invasive ventilation
SAPSII < 40
Inotropic and vasopressor support
SAPSII ≥ 50
Cardiac dysfunction / Circulatory failure
Non-invasive ventilation (BPAP)
Other organ dysfunction
Non-invasive ventilation (CPAP)
Respiratory organ dysfunction
SAPSII [40-49]
(B)
After matching
Before matching
